# Supplementary material for: Collaborative intelligence in AI: Evaluating the performance of a council of AIs on the USMLE
Source: PLOS Digit Health. 2025 Oct 9;4(10):e0000787. doi: 10.1371/journal.pdig.0000787 (PMC12510544; doi:10.1371/journal.pdig.0000787)
Supplement: S2 File — (ZIP) [file pdig.0000787.s002.zip › S2 Data Raw data files containing unprocessed question inputs and LLM outputs including transcripts of deliberations/STEP3_Question-62-1_Type-MC-NJ.docx]

**Facilitator** **[Round 1]:** A 71-year-old woman is brought to the emergency department by her daughter for evaluation of her mental status. The daughter says, ""I visited mom today for the first time in 6 months; her memory has worsened, her bills are unpaid, and her house is unusually messy."" The patient says, ""I'm perfectly healthy."" Medical history is significant for hypertension diagnosed more than 25 years ago, and two small strokes occurring 3 years and 7 months ago. The daughter gives you three empty medicine bottles from her mother's home: metoprolol, hydrochlorothiazide, and aspirin. The patient is 165 cm (5 ft 5 in) tall and weighs 59 kg (130 lb); BMI is 22 kg/m². Vital signs are temperature 36.4°C (97.6°F), pulse 76/min, respirations 16/min, and blood pressure 196/112 mm Hg. Pulse oximetry on room air shows an oxygen saturation of 93%. Muscle strength in her left hand is 1/5. Strength in the right extremities is 4/5. The patient is oriented only to self; she does not know the day of the week or today's date. She recalls 0 of 3 words at 5 minutes, and she is unable to name 2 objects, correctly draw interlocking pentagons, or count backwards by serial sevens. Complete blood count, renal function studies, liver function studies, and urinalysis are all within the reference ranges. ECG shows no abnormalities. Which of the following is the most likely underlying cause of the patient's symptoms? (A) Alzheimer disease (B) Amyotrophic lateral sclerosis (C) Cortical basal ganglionic degeneration (D) Neurosyphilis (E) Vascular dementia

**AI_A:** RESTATING THE QUESTION: The question concerns a 71-year-old woman who has been brought into the emergency room with changes in her mental status. She has recently demonstrated worsened memory and an inability to take care of usual tasks around the house. The patient has a medical history significant for hypertension and two small strokes, and reports being perfectly healthy. She shows signs of cognitive impairment, such as disorientation and memory failure. Her vital signs, blood work, and ECG are roughly normal despite high blood pressure. The question is asking us to figure out what the underlying cause of her symptoms might be. PLANNING: Let's start by identifying some key assumptions and considering alternative perspectives. The immediate assumption might be that these symptoms are due to progressive dementia, such as Alzheimer's disease, potentially as a result of her hypertension. However, her high blood pressure and history of two small strokes point towards the possibility of vascular dementia. As the patient's symptoms have apparently declined noticeably over six months, this also aligns more suitably with the often step-wised-progression associated with vascular dementia, rather than the slow, gradual decline typically seen in Alzheimer's. This reasoning hypothesis needs to be further evaluated clinically. CLINICAL REASONING: Alzheimer's disease is characterized by progressive memory loss and confusion. This patient's marked cognitive issues, including disorientation, memory deficits, and difficulties with complex tasks (drawing, counting backwards), might suggest such an illness. However, these symptoms alone don't confirm Alzheimer's disease. Meanwhile, vascular dementia is brought on by conditions that block or reduce blood flow to the brain, depriving brain cells of necessary oxygen and nutrients. Hypertensive vascular disease and a history of multiple strokes, as seen in this patient, are strong risk factors for vascular dementia. CONCLUDING: Given patient's history of hypertension and small strokes, her symptoms are more characteristically associated with vascular dementia than Alzheimer's disease or one of the other choices offered. RESPONSE: The most likely underlying cause of this patient's symptoms is vascular dementia (E).

**AI_C:** (1) RESTATING THE QUESTION: This clinical case highlights a 71-year-old woman, who is being brought in for a mental status check due to memory issues and an unusual pattern of messiness at home. Despite her daughter's concern and her significant medical history that includes hypertension and two strokes, the patient insists she's healthy. The terms: metoprolol, hydrochlorothiazide, and aspirin, could be recognized as medications commonly taken by patients with underlying cardiovascular diseases. Her physical examination presents weakness in her extremities, disorientation, poor recall, and difficulty with tasks that test cognitive function without any abnormalities in laboratory tests or ECG. With these pieces of information, we are challenged to identify the most likely underlying cause of the presented symptoms. (2) PLANNING: It can be assumed that these symptoms might be indicative of a neurological or cognitive disorder. By questioning this assumption, we're led to particular conditions such as Alzheimer's disease, Amyotrophic lateral sclerosis, Cortical basal ganglionic degeneration, Neurosyphilis, and Vascular dementia. To clinically prioritize these assumptions, we must consider conditions that align closely with the patient's present symptoms and history. (3) CLINICAL REASONING: Alzheimer's disease could be a plausible cause for memory loss and cognitive impairment; however, the patient's history of strokes and hypertension suggests a vascular cause. Amyotrophic lateral sclerosis primarily causes motor-neuron degeneration, but the patient's primary symptoms are cognitive. Cortical basal ganglionic degeneration typically presents with asymmetrically reduced movement and rigidity which are not primary concerns of this patient. Neurosyphilis could produce similar symptoms, but the patient has no other signs of syphilis and a more likely diagnosis can be made based on her medical history. (4) CONCLUDING: Given the fact that our patient has a history of hypertension and two strokes, with cognitive impairment and weakness being primary symptoms, Vascular dementia becomes a likely diagnosis. This condition can occur when reduced blood flow, often due to small vessel disease or strokes, deprives brain tissue of the oxygen and nutrients it needs for proper function. (5) RESPONSE: Thus, the most likely underlying cause of this patient's symptoms is Vascular dementia (E), given her history of hypertension and strokes along with her cognitive and physical decline.

**AI_B:** RESTATING THE QUESTION: We are dealing with an elderly woman who appears to be experiencing significant cognitive and motor decline, alongside other symptoms. Her medical history includes hypertension and prior strokes. We are being asked to identify the most probable cause of her symptoms from the options provided. PLANNING: Firstly, this looks primarily to be a process of differential diagnosis of neurodegenerative conditions. The given options include Alzheimer's disease, Amyotrophic lateral sclerosis (ALS), Cortical basal ganglionic degeneration (CBGD), Neurosyphilis, and Vascular dementia. Each of these entities present with unique signs and symptoms. Alzheimer's typically presents with a progressive loss of memory. ALS is primarily a motor neuron disease causing muscular weakness and atrophy. CBGD is a rare, neurodegenerative disease that presents with movement and cognitive disorders. Neurosyphilis, a tertiary stage of syphilis, can present with various neurological symptoms. Finally, vascular dementia is characterized by cognitive impairment due to ischemia or hemorrhages in the brain. CLINICAL REASONING: The patient shows signs of cognitive impairment as evidenced by her inability to recall words, name objects, correctly perform a drawing task, and perform backwards counting. She also has a history of hypertension and prior strokes. She also presents with muscle weakness. While any dementia would affect cognitive function, Alzheimer's disease typically does not present with such a marked degree of motor impairment. ALS could explain the motor impairment but does not align well with the cognitive deficits seen. The pattern of impaired cognition and motor dysfunction is not typical for CBGD. Neurosyphilis could theoretically explain the features, but there is little to suggest any prior history of syphilis or indeed symptoms specific to that condition. Vascular dementia, however, is a form of dementia caused by impairment in blood supply to the brain. Chronic hypertension, as seen in our patient, is a major risk factor for this. Moreover, the history of prior strokes and the current elevated blood pressure provides further support for this diagnosis. Additionally, the acute worsening could point towards a stepwise decline in cognition, again indicative of vascular dementia. CONCLUDING: Considering all these factors alongside the patient's history of hypertension, history of stroke and elevated blood pressure, the clinical picture fits most accurately with vascular dementia. RESPONSE: The most likely underlying cause of the patient's symptoms is (E) Vascular dementia.

**AI_E:** RESTATING THE QUESTION: We have a 71-year-old woman who has recently displayed noticeable cognitive impairment such as memory loss and disorientation, and physical impairment such as decreased muscle strength in one hand. She has a significant medical history of hypertension and two small strokes. Her blood analyses are within normal range and she shows no ECG abnormalities. Given her symptoms and medical history, we need to figure out the most likely root cause of both her cognitive and physical symptoms. The options presented include (A) Alzheimer's disease, known for causing progressive dementia; (B) Amyotrophic lateral sclerosis, a neurodegenerative disease characterized by motor neuron decline; (C) Cortical Basal Ganglionic Degeneration, a rare neurodegenerative disease affecting cognition and motor skills; (D) Neurosyphilis, a bacterial infection of the brain or spinal cord; and (E) Vascular dementia, cognitive impairment due to reduced blood flow in the brain. PLANNING: The assumption here is that one of these listed conditions is the primary cause and driver of the patient's symptoms. We may evaluate this assumption by correlating each listed condition with the observed medical history and symptoms. For instance, we note that Alzheimer disease primarily targets the brain, causing progressive cognitive decline, but less motor impairment which is evident in the case of this patient. ALS, on the other hand, is a neurodegenerative disorder that primarily affects motor neurons with less cognitive involvement. CLINICAL REASONING: The patient's history of long-standing hypertension and two small strokes are significant pieces of information. They suggest the potential for vascular damage within the brain, which may contribute to cognitive disruption and physical debilities. Long-term hypertension can result in microvascular damage, leading to chronic ischemic changes in the brain. Two small strokes suggest the potential for multi-infarct related impairment, pointing towards a likely diagnosis of vascular dementia (a multi-infarct dementia). On the other hand, her impaired cognitive state and motor dysfunction, specifically disorientation and weakness in one hand, resonate more with symptoms encountered with vascular dementia. Progressive confusion, poor concentration, impaired judgment, restlessness, gait disturbances are common in vascular dementia. CONCLUDING: The phenomenology of her past medical history, namely, systemic hypertension and infarcts, along with cognitive and physical decline – disorientation, decreased motor strength – makes the argument strong for diagnosing vascular dementia. Moreover, the other options, although they explain some of the symptoms, do not convincingly cover the overall presentation as well as vascular dementia does. RESPONSE: Therefore, considering her clinical presentation, comprehensive history, and the chronicity of symptoms, (E) Vascular dementia is the most likely underlying cause of the patient's symptoms.

**AI_D:** RESTATING THE QUESTION: The question presents us with a 71-year-old lady who was brought by her daughter to the emergency department due to concerns about her changed mental state. The patient has shown worsening memory, neglect of her household responsibilities, and a messy household. The patient refuses that anything is wrong with her health. Her history indicates hypertension for over two decades and two mild strokes in the past. She has been prescribed metoprolol, hydrochlorothiazide, and aspirin. The patient has asymmetric weakness in her extremities and her cognitive orientation and memory seem impaired. Laboratory blood tests and an EKG are normal. The question wants an accurate identification of the probable rationale behind the patient's symptoms, choosing from Alzheimer's disease, amyotrophic lateral sclerosis, cortical basal ganglionic degeneration, neurosyphilis, and vascular dementia. PLANNING: Considering the patient's symptoms, history and diagnostic results, the assumption is that her altered mental status is because of a neurological or mental health disorder. These symptoms can be attributed to Alzheimer's disease, neurosyphilis, vascular dementia, cortical basal ganglionic degeneration, or amyotrophic lateral sclerosis. However, paired with her medical history, the likelihood of each condition varies. For instance, Alzheimer's disease is usually accompanied by slower deterioration and not typically accompanied by prominent hypertension-associated symptoms. This indicates the necessity to query the assumption that her mental changes are purely due to a progressive neurodisease like Alzheimer's or cortical basal ganglionic degeneration. Another assumption requiring questioning is whether the presentation of symptoms is due to untreated hypertension, resulting in cerebrovascular changes like vascular dementia. CLINICAL REASONING: The patient has a known history of hypertension, which, if untreated over a long period, can lead to chronic damage to the blood vessels supplying the brain leading to cerebrovascular accidents ("strokes"). This history along with her symptoms of progressive memory loss and changes in normal household routines, as well as cognitive defects indicated by her inability to recall words or draw objects, strongly suggest that the patient is suffering from a form of dementia. However, her asymmetric weakness, particularly in the left hand, suggests a vascular etiology rather than a diffuse degenerative condition like Alzheimer’s disease. Given her history of hypertension and previous stroke events, it is probable that the ongoing hypertensive damage to her vessels has resulted in numerous tiny infarcts, a condition described as vascular dementia." CONCLUDING: In vascular dementia, the thinking process has been damaged by a series of small strokes or significant changes to the brain’s blood vessels. These changes disrupt the brain’s ability to perform cognitive functions like thinking, planning, reasoning, and memory. In this patient's case, the combination of progressive memory loss and cognitive impairment, history of hypertension and small strokes, as well as the absence of other systemic anomalies, makes vascular dementia the most credible diagnosis. RESPONSE: The most probable cause that can explain the patient's symptoms is (E) Vascular dementia.

**Facilitator** The consensus statement synthesizes the responses of AI_A, AI_B, AI_C, AI_D, and AI_E as follows: The question presents a 71-year-old woman with a significant medical history of hypertension and two small strokes, who has recently shown cognitive and physical impairment. Her symptoms include memory loss, disorientation, and decreased muscle strength in one hand. Despite her insistence on being healthy, her daughter reports a noticeable decline in her ability to manage her household and personal affairs. Her vital signs, blood work, and ECG are roughly normal, except for high blood pressure. The task is to identify the most likely underlying cause of her symptoms from the options provided: Alzheimer's disease, Amyotrophic lateral sclerosis (ALS), Cortical basal ganglionic degeneration (CBGD), Neurosyphilis, and Vascular dementia. The immediate assumption might be that these symptoms are due to a progressive neurodegenerative condition such as Alzheimer's disease. However, Alzheimer's typically presents with a slow, gradual decline, which does not align with the patient's relatively rapid deterioration over six months. Moreover, Alzheimer's disease does not typically present with such a marked degree of motor impairment. ALS and CBGD, while they could explain the motor impairment, do not align well with the cognitive deficits seen in the patient. Neurosyphilis could theoretically explain the features, but there is little to suggest any prior history of syphilis or indeed symptoms specific to that condition. On the other hand, the patient's history of hypertension and prior strokes, along with her cognitive and physical symptoms, strongly suggest a vascular cause. Vascular dementia is a form of dementia caused by conditions that block or reduce blood flow to the brain, depriving brain cells of necessary oxygen and nutrients. Hypertensive vascular disease and a history of multiple strokes, as seen in this patient, are strong risk factors for vascular dementia. The acute worsening could point towards a stepwise decline in cognition, again indicative of vascular dementia. In conclusion, given the patient's history of hypertension and small strokes, her symptoms are more characteristically associated with vascular dementia than Alzheimer's disease or one of the other choices offered. Therefore, the most likely underlying cause of this patient's symptoms is Vascular dementia (E).
